# Analysis of the Correlation between Eating Away from Home and BMI in Adults 18 Years and Older in China: Data from the CNNHS 2015

**DOI:** 10.3390/nu14010146

**Published:** 2021-12-29

**Authors:** Xiaoqi Wei, Dongmei Yu, Lahong Ju, Xue Cheng, Liyun Zhao

**Affiliations:** Chinese Center for Disease Control and Prevention, Laboratory of Trace Element Nutrition of National Health Commission, National Institute for Nutrition and Health, Beijing 100050, China; XQ437073568@163.com (X.W.); yudm@ninh.chinacdc.cn (D.Y.); julh@ninh.chinacdc.cn (L.J.); chengxue@ninh.chinacdc.cn (X.C.)

**Keywords:** eat away from home, frequency, BMI, adults, China

## Abstract

The purpose of this study is to examine the relationship between the frequency of eating away from home (EAFH) and Body mass index (BMI) in adults. The data were collected from 2015 China Adult Chronic Disease and Nutrition Surveillance (CNNHS 2015). Adults aged 18 and above who had complete dietary frequency questionnaire data were recruited as the research objects. The frequency of EAFH among different genders and BMI groups were compared, and multiple linear regression method was used to analyze the correlation between frequency of EAFH and BMI of adults aged 18 years and above with different gender, age, family per capita annual income, education level, marital status, and occupation level. The frequency of EAFH was higher for adults aged 18–44, eastern region, urban, family per capita annual income of 20,000 RMB or more, highly educated, unmarried, school students, employed people, and obese adults, which were 3.64, 3.30, 3.71, 4.30, 5.92, 5.64, 9.29 and 2.68 times per week, respectively. The highest frequency of EAFH was obese men in urban areas aged 18–44 years, which was 5.63 times per week. Multiple linear regression analysis showed that the frequency of EAFH for breakfast was not associated with BMI (*p* > 0.05), the frequency of EAFH for lunch was negatively correlated with BMI (*β* = −0.024, *p* = 0.008), and the frequency of EAFH for dinner was positively correlated with BMI (*β* = 0.040, *p* = 0.004).The frequency of EAFH of male (*β* = 0.013, *p* = 0.008), 60 years old and above (*β* = 0.022, *p* = 0.021), family per capita annual income less than 10,000 RMB (*β* = 0.019, *p* = 0.005), junior high school education or below (*β* = 0.012, *p* = 0.009), and unemployed/retired (*β* = 0.029, *p* = 0.003) adults were positively correlated with BMI. While for women, the frequency of EAFH (*β* = −0.019, *p* = 0.001) was negatively correlated with BMI. In terms of frequency of EAFH for breakfast, female (*β* = 0.027, *p* = 0.041), people aged 45–59 years (*β* = 0.042, *p* = 0.002), aged 60 and above (*β* = 0.047, *p* = 0.017), eastern China (*β* = 0.034, *p* = 0.010), junior high school education or below (*β* = 0.045, *p* = 0.001), married/cohabiting (*β* = 0.024, *p* = 0.008) adults’ frequency of EAFH for breakfast of was positively correlated with BMI. In terms of frequency of EAFH for lunch, female (*β* = 0.056, *p* = 0.001), people aged 45–59 years (*β* = 0.024, *p* = 0.005), eastern China (*β* = 0.034, *p* = 0.004), rural areas (*β* = 0.035, *p* = 0.006), moderate and high family per capita annual income (*β* = 0.043, *p* = 0.007; *β* = 0.029, *p* = 0.039), high education level (*β* = 0.039, *p* = 0.034), married/cohabiting (*β* = 0.028, *p* = 0.001), on-the-job personnel (*β* = 0.033, *p* = 0.001) frequency of EAFH for lunch were negatively correlated with BMI. In terms of the frequency of EAFH for dinner, the frequency of EAFH for dinner had a significant positive influence on the BMI of males (*β* = 0.061, *p* = 0.001). The frequency of dinner EAFH for 18–44 years old (*β* = 0.042, *p* = 0.028), central region (*β* = 1.000, *p* < 0.001), rural areas (*β* = 0.055, *p* = 0.013), married/cohabiting (*β* = 0.048, *p* = 0.001), on-the-job personnel (*β* = 0.037, *p* = 0.035) adults were positively correlated with BMI. The frequency of EAFH in urban obese men aged 18–44 was the highest. The frequency of EAFH for breakfast was not correlated with BMI, the frequency of EAFH for lunch was negatively correlated with BMI, and the frequency of EAFH for dinner was positively correlated with BMI. The analysis between EAFH according to the current definition and health-related outcomes is mixed. It is suggested that relevant authorities redefine EAFH from the perspective of health outcomes.

## 1. Introduction

In the past decade, the adults’ body mass index (BMI) and obesity rate in China has shown a rapid growth trend. From 2004 to 2018, the average BMI of Chinese adults increased from 22.7 kg/m^2^ to 24.4 kg/m^2^. The obesity rate increased from 3.1% to 8.1%, and the average annual BMI increased by 0.09 kg/m^2^ from 2010 to 2018 [1,2]. Unhealthy eating behavior includes skipping breakfast, night eating, eating away from home (EAFH), and emotional eating [3,4,5]. The large increase in the frequency of EAFH is a prominent feature of the change of eating behavior in the world. The NHANES showed that the proportion of people EAFH was 34% from 2005 to 2014. The proportion of people aged 20 and over EAFH was 64% from 2017 to 2018 [6,7]. In the UK, the proportion of adults aged 19 and above EAFH was 27.1% from 2008 to 2012, among which the proportion of takeaway at least once a week was 21.1% [8]. The proportion of Japanese adults aged 20 and older who EAFH at least once a week rose to 33.6% in 2019 from 32.3% in 2015 [9]. The proportion of Chinese adults aged 18–44 and 45–59 years who EAFH in a week rose from 19.5% and 11.1% in 2002 to 41.3% and 24.3% in 2015, respectively [10].

EAFH not only brings convenience to people but also causes many health problems. A number of studies have shown that EAFH is an important reason for the increase in body weight, BMI and waist circumference [11,12,13], especially the intake of dietary energy from EAFH is higher. In addition, the EAFH diet includes a wider variety of foods but tends to be energy-dense food [14]. Eating too much energy-dense food can lead to an increase in body fat, which in turn increases the risk of overweight and obesity [15,16]. The study of Zhang [16] found that the risk of overweight and obesity was 1.53 times higher for men who EAFH three times or more per week than for those who did not EAFH, and women who EAFH one to two times a week and three or more times a week had 1.60 times and 2.23 times higher risk of being overweight and obese than those who did not eat out, respectively Foreign studies [15] found that BMI increased by 0.8 kg/m^2^ and 0.6 kg/m^2^ for each additional meal in fast food restaurants and restaurants, respectively, and the total number of meals EAFH more than seven times per week would increase BMI and obesity risk [17]. According to the 2015 Survey on Nutrition and Health status of Chinese residents, the risk of obesity among male residents aged 18–59 who EAFH 14–21 times/week is 1.8 times that of the non-EAFH group [10].

Our study analyzed the correlation between frequency of EAFH and BMI and its influence factors by using the dietary data from the China National Chronic Non-communicable Disease and Nutrition Surveillance of adults in 31 provinces of China from 2015 (CNNHS 2015) of the Former National Health Commission of the People’s Republic of China and Chinese Center for Disease Control and Prevention, to provide the basis for our adult dining out and health-related risk control strategy.

## 2. Materials and Methods

### 2.1. Study Design and Samples

Multistage stratified random sampling method was used to collect data from 302 monitoring points of CNNHS 2015 [18]. After data cleaning, national dietary surveys conducted by 77,944 adults aged 18 years and older were used. The project was reviewed by the Ethical Review Committee of the Chinese Center for Disease Control and Prevention (201519-B). All respondents signed in-formed consent before the investigation.

### 2.2. Data Collection and Measurements

Data were collected from the unified basic information registration of family members and food Frequency Questionnaire (FFQ) in CNNHS 2015 and were collected by trained and qualified investigators through face-to-face interviews. Physical measurements include height and body weight. Height and weight were measured by investigators using standardized equipment. Height was measured with a height meter (TZG type) with a minimum scale of 0.1 cm. Weight was measured with an electronic scale (Tanita HD-390) with a minimum scale of 0.1 kg.

### 2.3. Quality Assurance

The monitoring adopts three unified criteria, including unified investigation methods, unified equipment, and quality control, investigators who have been unified trained and assessed. Data were double input, and the national working group uniformly cleaned up and reviewed the data reported by each province.

### 2.4. Assessment of EAFH

The questionnaire related to our study included the frequency of breakfast, lunch, and dinner at different dining locations over the past week (7 days). “Frequency of EAFH” was defined as the respondents eating at least once outside in the past 7 days or eating non-homemade food as regular meals. Dining places are divided into seven ways: home, buy home eating (takeout, order, and box lunch), workplace/school dining hall, a Chinese restaurant (including fast food restaurant), Western restaurant (including fast food restaurant), bakery/cake shop/coffee shop, and other places.

### 2.5. Covariates

A wide range of potential confounders were accounted for: (1) Body mass index (BMI) was calculated with the formula: body weight (kg)/height^2^ (m^2^), According to the Criteria of Weight for Adults of the health industry standard of China, WS/T 428-2013, BMI was categorized as underweight (<18.5 kg/m^2^), normal (18.5 kg/m^2^≤ to <24 kg/m^2^), overweight (24 kg/m^2^≤ to <28 kg/m^2^), obese (≥28 kg/m^2^); (2) age was categorized as 18–44 years, 45–59 years, ≥60years; (3) education level was categorized as low (junior high and below), moderate (high school/technical secondary school/technical school) or high (junior college and above); (4) Family per capita annual income was categorized as low (<10,000 RMB), moderate (10,000 RMB≤ to <20,000 RMB), high (≥20,000 RMB) or Unclear/unknown. (5) Marital status was categorized as unmarried, married/cohabiting, divorced/widowed; (6) Occupations are classified into Farming and aquaculture (including agriculture, forestry, animal husbandry, fishery, and water conservancy), other occupational people, school students, retired/unemployed.

### 2.6. Statistical Analysis

Data cleaning and analysis were performed using SAS 9.4 software (SAS Institute Inc., Cary, NC, USA). To study the distribution of the frequency of EAFH among adults of different genders and BMI groups based on the weekly frequency of EAFH obtained from the FFQ questionnaire, an ANOVA test was used for comparison between different groups. The multiple linear regression model was established by the Ordinary Least Square (OLS) method, taking gender, age, region, urban/rural, family per capita annual income, education level, marital status, and occupational level as independent variables with BMI as the dependent variable. In addition, considering the possible influence of EAFH time (breakfast, lunch, or dinner) on BMI, the EAFH time was included in the model for analysis, and the heteroscedasticity Robust estimator was obtained by Robust standard deviation estimation. *p* < 0.05 was considered statistically significant.

## 3. Results

### 3.1. The Frequency of Eating Away from Home (EAFH) in Different Genders

A total of 77,944 adults aged 18 years and older participated in the study, including 36,836 males, 41,108 females. In 2015, the average frequency of Chinese adults aged 18 and above EAFH was 2.59 times per week. 18–44 years old, eastern, urban, family per capita annual income ≥20,000 RMB, college and above, unmarried, school students, obese adults have a higher frequency of EAFH, 3.64, 3.30, 3.71, 4.30, 5.92, 5.64, 9.29, and 2.68 times per week, respectively.

In terms of gender, the average frequency of EAFH for men was 3.23 times per week. The average frequency of EAFH for women was 1.92 times per week. The frequency of EAFH per week for men was higher than that of women and the difference was statistically significant (*p* < 0.05). Aged 18–44, eastern, urban, family per capita annual income ≥20,000 RMB, college or above, unmarried, school students, and obese adult males had a higher frequency of EAFH, which was 4.41, 3.99, 4.38, 5.08, 6.48, 5.35, 9.09, 3.93 times per week, respectively, and male frequency of EAFH is higher than the female frequency of EAAFH, the difference is statistically significant except for school students (*p* < 0.05). The results are detailed in Table 1.

The average frequency of EAFH per week in underweight, normal weight, overweight and obese groups was 2.83, 2.67, 2.40, and 2.68 times, respectively. In terms of gender, the average frequency of EAFH per week was higher in the obese male group (3.93 times, *p* < 0.05). The average frequency of EAFH per week was higher in the underweight female group (2.76 times, *p* < 0.05). In terms of age, obese adults aged 18–44 had the highest frequency of EAFH, reaching 3.83 times per week (*p* < 0.05). In terms of regional distribution, obese males had the highest frequency of EAFH among urban adults aged 18–44, followed by underweight males, which were 5.63 and 5.44 times per week, respectively. From the family per capita annual income, education level, and career classification, the underweight and obese groups were the highest in both the college and above education groups and the single/unmarried group. However, the underweight and normal weight groups were the highest in the group of family per capita annual income ≥20,000 RMB. The normal weight and obese group were the highest in the unemployed/retired group. The results are detailed in Table 1, Table A1, Table A2 and Table A3.

### 3.2. Association between Eating Away from Home (EAFH) and BMI

In 2015, the frequency of Chinese adults aged 18 and above EAFH for breakfast, lunch, and dinner was 0.95, 1.23, and 0.42 times per week. For males, the frequency of EAFH for breakfast, lunch. And dinner was 1.14, 1.55, and 0.53 times per week, respectively. For females, the frequency of EAFH for breakfast, lunch, and dinner was 0.74, 0.88, and 0.29 times per week, respectively. The frequency of EAFH for breakfast and lunch was higher in the underweight group (1.12 times and 1.29 times per week, respectively), while the frequency of EAFH for dinner was higher in the obese group (0.45 times per week).

Multiple linear regression was used to analyze the relationship between frequency of EAFH and BMI of adults with different gender, age, family per capita annual income, education level, marital status, and occupation level, and the results were as follows.

For frequency of EAFH, there was no correlation between adults’ frequency of EAFH and BMI (*p* > 0.05), while after grouping by gender, men’s frequency of EAFH was positively correlated with BMI (*β* = 0.013, *p* = 0.008), while women’s frequency of EAFH was negatively correlated with BMI (*β* = −0.019, *p* = 0.001). In both urban and rural areas, there was no correlation between frequency of EAFH and BMI of urban and rural adults (*β* = 0.009, *p* > 0.05; *β* = 0.006, *p* > 0.05). For age segments, the frequency of EAFH in adults aged 60 years and older was positively associated with BMI (*β* = 0.022, *p* = 0.021). In terms of family per capita annual income, adults’ frequency of EAFH with family per capita annual income below 10,000 RMB showed a positive correlation with BMI (*β* = 0.019, *p* = 0.005). As far as the level of education, the frequency of EAFH for adults with junior high school and below showed positive frequency with BMI (*β* = 0.012, *p* = 0.009); In terms of occupation, unemployed/retired adults’ frequency of EAFH was positively correlated with BMI (*β* = 0.029, *p* = 0.003).

In terms of frequency of EAFH for breakfast, female (*β* = 0.027, *p* = 0.041), 45–59 years old (*β* = 0.042, *p* = 0.002), 60 years old and above (*β* = 0.047, *p* = 0.017), eastern China (*β* = 0.034, *p* = 0.010), junior high school and below (*β* = 0.045, *p* = 0.001), married/cohabiting adults (*β* = 0.024, *p* = 0.008) frequency of EAFH for breakfast was positively correlated with BMI.

In terms of the frequency of EAFH for lunch, the frequency of EAFH for lunch was negatively correlated with BMI of Chinese adults in 2015 (*β* = −0.024, *p* = 0.004), while there was no correlation between the frequency of EAFH for lunch and BMI of adult males (*β* = −0.005, *p* > 0.05), the frequency of EAFH for lunch was negatively correlated with BMI in adult women (*β* = −0.056, *p* < 0.001). 45 to 59 years old (*β* = 0.024, *p* = 0.05), eastern China (*β* = 0.034, *p* = 0.004), countryside (*β* = 0.035, *p* = 0.006), moderate and high family per capita annual income (*β* = 0.043, *p* = 0.007; *β* = 0.029, *p* = 0.039), junior college and above (*β* = 0.039, *p* = 0.034), married/cohabiting *β* = 0.028, *p* = 0.001), on-the-job personnel (*β* = 0.033, *p* = 0.001) frequency of EAFH for lunch was negatively correlated with BMI.

In terms of the frequency of EAFH for dinner, there was a positive correlation between the frequency of EAFH for dinner and BMI of Chinese adults (*β* = 0.040, *p* = 0.004), and there was a significant positive correlation between the frequency of EAFH for dinner and BMI of adult males (*β* = 0.061, *p* = 0.001), the frequency of EAFH for dinner was not associated with BMI in adult females (*β* = −0.025, *p* > 0.05). 18–44 years old (*β* = 0.042, *p* = 0.028), central region (*β* =1.000, *p* < 0.001), rural (*β* = 0.055, *p* = 0.013), married/cohabiting (*β* = 0.048, *p* = 0.001), and employed people’s frequency of EAFH for dinner of were positively correlated with BMI (*β* = 0.037, *p* = 0.035). The results are shown in Table 2.

## 4. Discussion

The study explored the relationship between the frequency of EAFH (include the frequency of EAFH for breakfast, lunch, and dinner) and BMI using data from 77,944 Chinese adults aged 18 and older from the 2015 China National Chronic Non-communicable Disease and Nutrition Surveillance. The results showed that males aged 18–44, eastern, urban, family per capita annual income ≥20,000 RMB, junior college or above, unmarried, school students, other occupational people, and obese people had a higher frequency of EAFH. The frequency of EAFH was related to BMI at different levels such as genders, ages, regions, urban or rural, family per capita annual income, education level, and occupation level. However, gender, age, family per capita annual income, marital status, education, and career levels, dining place, and time of EAFH were all influencing factors of BMI, which is consistent with many other findings [12,13,18,19].

In terms of the frequency of EAFH for breakfast, there was a positive correlation between the frequency of EAFH for breakfast and BMI for women aged 45 and over. Perhaps because most of these people are married women, and they tend to have time to make substantial breakfasts at home or buy them from breakfast shops then eat them at home. Moreover, Breakfast in China is diversified, especially with distinct regional characteristics, such as fried breadsticks and fried pancakes in the north and steamed dumplings in the south, which are often convenient to eat but low in nutritional value. These foods are high in calories, and excessive consumption may lead to obesity [12,20]. In terms of the frequency of EAFH for lunch, the frequency of EAFH for lunch for men is not correlated with the increase of BMI, while the frequency of EAFH for lunch for women is negatively correlated with BMI. Tian et al. [12] obtained the same results as this study, which may be related to the fact that adult male workers usually have lunch at the workplace or nearby. Lassen’s intervention study [21] showed that meal patterns in workplaces or group canteens may be more reasonable than those in restaurants or takeaways. Roos’ study [22] also showed that the Chinese adult male lunch in the workplace canteen is related to a decrease in BMI, which may be that the workplace canteen generally provides relatively balanced food for employees to choose from and will eat more vegetables for lunch. In terms of the frequency of EAFH for dinner, the frequency of EAFH for dinner for adults aged 18–44 is positively correlated with BMI, which may be that most people in this age group are occupational staff, and the frequency of EAFH for dinner for occupational people is also positively correlated with BMI. The frequency of EAFH for dinner in adult men is significantly correlated with the increase of BMI, which is consistent with the results of foreign studies [12], which may be related to the fact that men usually eat in restaurants or fast-food restaurants for social activities [23]. This study also found that school students are one of the main groups with a high frequency of EAFH, which may be due to the high proportion of students having meals in school canteens [24], but it has been proved that school is an important environment to provide healthy food and promote students’ healthy diet [25]. Studies that investigated the density of fast food restaurants and restaurants near residents to investigate the relationship between EAFH and BMI, the results found that the increased density of fast food restaurants is associated with increased BMI, and the density of other restaurants or canteens is negatively associated with BMI, it may be with fast food is mainly fried cooking methods, which increases fat intake and the risk of overweight and obesity [23,26,27]. Therefore, choosing different places to EAFH has different effects on BMI regardless of when EAFH. The reason for the difference may be the unreasonable definition of EAFH. The definition of dining out now used in domestic and foreign studies is all places except families, which includes restaurants for profit, snack bars/stalls, convenience stores and fast food, and also includes nonprofit purpose workplace or collective canteens [23,28,29]. Therefore, our study suggests that the current definition of EAFH is mixed in the analysis of EAFH and health-related outcomes, such as the diversity of food supply institutions, which should separate workplace or collective dining halls from profit dining such as restaurants. Simply incorporating all out-of-home eating behaviors into EAFH has obvious confusion in definition, which covers up and underestimates the health effects of unreasonable eating patterns in restaurants and other dining places. It is recommended that the relevant authorities redefine EAFH from the perspective of influencing health outcomes.

The study analyzed the correlation between the frequency of EAFH, as well as the frequency of EAFH for breakfast, lunch, and dinner, and BMI of Chinese adults aged 18 and over in 2015. The study analyzed the possible influence of the frequency of EAFH on overweight and obesity and the insufficiency of the current definition of EAFH to provide a scientific basis for the development of health risk control measures for Chinese adults eating out of home. However, the study still has the following limitations and needs further development. First, the effect of dietary intake and physical activity level on BMI was not considered. Secondly, this study is a cross-sectional study and cannot conclude a causal relationship between the frequency of EAFH and BMI. For example, the current diet may not be representative of previous years’ diets, which is the diet that led to obesity, not the current diet. Thus, a prospective study, which will be considered in combination with food intake, physical activity levels, is needed in the subsequent studies.

## 5. Conclusions

The frequency of EAFH in urban obese men aged 18–44 was the highest. The frequency of EAFH for breakfast was not correlated with BMI, the frequency of EAFH for lunch was negatively correlated with BMI, and the frequency of EAFH for dinner was positively correlated with BMI. The analysis between EAFH according to the current definition and health-related outcomes is mixed. It is suggested that relevant authorities redefine EAFH from the perspective of health outcomes.

## Figures and Tables

**Table 1:** Frequencies of EAFH of different gender categories.

	Total		Men		Women		F
	*n*	E AFH	*n*	E AFH	*n*	E AFH	
Total	77,944	2.59 (0.11)	36,836	3.23 (0.12)	41,108	1.92 (0.10)	1496.39 *
Age Group, year							
18–44	23,070	3.64 (0.09)	10,416	4.41 (0.14)	12,654	2.82 (0.10)	502.07 *
45–59	28,752	1.79 (0.05)	13,277	2.42 (0.08)	15,475	1.12 (0.05)	819.67 *
≥60	26,122	0.51 (0.02)	13,143	0.69 (0.03)	12,979	0.34 (0.02)	172.89 *
Area of the country							
East	29,502	3.30 (0.10)	13,864	3.99 (0.15)	15,638	2.55 (0.12)	581.25 *
Central	22,065	2.22 (0.07)	10,482	2.89 (0.12)	11,583	1.52 (0.07)	505.84 *
West	26,377	1.88 (0.06)	12,490	2.38 (0.10)	13,887	1.36 (0.07)	393.67 *
Residence location							
Urban	31,565	3.71 (0.09)	14,558	4.38 (0.14)	17,007	2.99 (0.11)	538.79 *
Rural	46,379	1.41 (0.04)	22,278	1.97 (0.07)	24,101	0.85 (0.04)	1063.14 *
Household Income Level							
Low	29,635	1.56 (0.06)	14,150	2.02 (0.10)	15,485	1.09 (0.08)	408.32 *
Moderate	19,042	2.61 (0.10)	8959	3.34 (0.17)	10,083	1.82 (0.09)	517.43 *
High	16,704	4.30 (0.13)	7911	5.08 (0.21)	8793	3.43 (0.16)	366.71 *
Unclear/unknown	12,563	2.20 (0.11)	5816	2.73 (0.14)	6747	1.70 (0.17)	163.74 *
Education Level							
Low	62,007	1.57 (0.04)	28,169	2.16 (0.07)	33,838	1.02 (0.04)	1382.76 *
Moderate	10,142	3.69 (0.16)	5759	4.11 (0.23)	4383	3.04 (0.18)	99.09 *
High	5795	5.92 (0.20)	2908	6.48 (0.29)	2887	5.29 (0.27)	57.49 *
Marital Level							
Spinsterhood	2914	5.64 (0.29)	1893	5.35 (0.37)	1021	6.24 (0.46)	12.23 *
Married/cohabitation	71,450	2.24 (0.04)	33,842	2.88 (0.07)	37,608	1.60 (0.04)	1555.16 *
Widowed/divorce/separation	3580	1.26 (0.17)	1101	2.25 (0.42)	2479	0.71 (0.09)	162.01 *
Employment							
Farming and aquaculture	35,358	0.93 (0.04)	17,624	1.34 (0.07)	17,734	0.50 (0.03)	715.35 *
Others	20,927	4.81 (0.10)	12,076	5.06 (0.14)	8851	4.42 (0.13)	61.38 *
Student	237	9.29 (0.80)	125	9.09 (0.88)	112	9.65 (1.56)	0.31
Unemployed/retired	21,422	0.86 (0.04)	7011	1.13 (0.08)	14,411	0.73 (0.05)	101.09 *
BMI							
Underweight	3002	2.83 (0.25)	1303	2.90 (0.43)	1699	2.76 (0.29)	0.60
Normal	36,686	2.67 (0.09)	17,465	3.13 (0.15)	19,221	2.23 (0.10)	311.79 *
Overweight	27,177	2.40 (0.07)	13,139	3.12 (0.10)	14,038	1.56 (0.08)	814.69 *
Obesity	11,079	2.68 (0.12)	4929	3.93 (0.19)	6150	1.28 (0.11)	881.93 *

Data are presented as $N\;or\;\bar{x}$(s). * *p* < 0.05 compared with women.

**Table 2:** Association between eating away from home (EAFH) and BMI.

Variable	EAFH		EAFH-Breakfast		EAFH-Lunch		EAFH-Dinner	
	Coefficient	Robust S.E.	Coefficient	Robust S.E.	Coefficient	Robust S.E.	Coefficient	Robust S.E.
Total	0.005	0.004	0.017	0.009	−0.024	0.008 *	0.04	0.014 *
Sex								
men	0.013	0.005 *	0.006	0.011	−0.005	0.011	0.061	0.018 *
women	−0.019	0.006 *	0.027	0.013 **	−0.056	0.013 *	−0.025	0.021
Age Group, year								
18–44	0.001	0.006	−0.006	0.013	−0.015	0.013	0.043	0.019 *
45–59	0.008	0.006	0.042	0.014 **	−0.024	0.012 *	0.016	0.022
≥60	0.022	0.009 *	0.047	0.020 *	0.002	0.023	0.007	0.038
Area of the country								
East	0.003	0.006	0.034	0.013 **	−0.034	0.012 **	0.030	0.020
Central	0.007	0.007	−0.011	0.016	−0.025	0.016	0.100	0.027 **
West	0.009	0.007	0.005	0.017	0.024	0.018	−0.011	0.027
Residence location								
Urban	0.009	0.005	0.018	0.010	−0.007	0.011	0.023	0.018
Rural	0.006	0.006	0.023	0.016	−0.035	0.013 **	0.055	0.022 *
Family per capital annual income								
Low	0.019	0.007 **	0.024	0.018	0.022	0.017	0.008	0.026
Moderate	0.001	0.008	0.025	0.017	−0.043	0.016 **	0.050	0.028
High	−0.009	0.007	0.001	0.014	−0.029	0.014 *	0.017	0.023
Unclear/unknown	0.020	0.010 *	0.033	0.025	−0.030	0.023	0.093	0.041 *
Education Level								
Low	0.012	0.005 **	0.045	0.011 **	−0.019	0.011	0.020	0.018
Moderate	0.000	0.009	−0.017	0.019	−0.003	0.019	0.036	0.032
High	−0.011	0.009	−0.010	0.019	−0.039	0.019 *	0.049	0.032
Marital Level								
Spinsterhood	−0.020	0.014	−0.062	0.036	0.004	0.034	0.002	0.046
Married/cohabitation	0.007	0.004	0.024	0.009 **	−0.028	0.009 **	0.048	0.015 **
Widowed/divorce/separation	0.005	0.024	−0.003	0.048	0.054	0.064	−0.069	0.100
Employment								
Farming and aquaculture	0.014	0.007	0.005	0.019	0.001	0.017	0.049	0.029
Others	−0.001	0.005	0.015	0.011	−0.033	0.010 **	0.037	0.017 *
Student	−0.043	0.032	−0.020	0.165	0.049	0.125	−0.159	0.172
Unemployed/retired	0.029	0.010 **	0.035	0.018	0.040	0.024	−0.002	0.039

Notes: BMI is the ratio of weight divided by square of height (kg/m^2^). Robust S.E. refers to the Robust standard error, * *p* < 0.01; ** *p* < 0.05. Multiple linear Regression analysis was performed for each variable separately, while controlling for other factors.

## Data Availability

The data presented in this study are non-public.

## Program

China Adults Chronic Diseases and Nutrition Surveillance of (2015).

## Appendix A

**Table A1:** Frequencies of EAFH of different BMI categories.

Variable	Underweight		Normal		Overweight		Obesity		F
	*n*	EAFH	*n*	EAFH	*n*	EAFH	*n*	EAFH	
Total	3002	2.83 (0.26)	36,686	2.67 (0.15)	27,177	2.40 (0.10)	11,079	2.68 (0.13)	13.25 *
Sex									
Men	1303	2.90 (0.47)	17,465	3.13 (0.19)	13,139	3.12 (0.14)	4929	3.93 (0.19)	68.31*
Women	1699	2.76 (0.30)	19,221	2.23 (0.14)	14,038	1.56 (0.09)	6150	1.28 (0.12)	406.20 *
Age Group, year									
18–44	1143	3.65 (0.39)	11,917	3.72 (0.21)	6961	3.40 (0.16)	3049	3.83 (0.21)	0.30
45–59	659	1.51 (0.30)	12,455	1.66 (0.08)	11,117	1.90 (0.09)	4521	1.86 (0.17)	19.24 *
≥60	1200	0.23 (0.05)	12,314	0.48 (0.03)	9099	0.60 (0.04)	3509	0.50 (0.05)	15.80 *
Area of the country									
East	1058	3.30 (0.48)	12,980	3.66 (0.29)	10,718	2.96 (0.17)	4746	2.94 (0.20)	84.85 *
Central	796	2.61 (0.55)	10,388	2.13 (0.17)	7823	2.10 (0.16)	3058	2.63 (0.24)	8.51 *
West	1148	2.31 (0.34)	13,318	1.81 (0.13)	8636	1.80 (0.15)	3275	2.19 (0.23)	2.09
Residence location									
Urban	957	3.80 (0.49)	13,410	3.94 (0.25)	11,949	3.43 (0.16)	5249	3.62 (0.20)	28.41 *
Rural	2045	1.76 (0.24)	23,276	1.43 (0.09)	15,228	1.28 (0.08)	5830	1.52 (0.12)	4.54 *
Household Income Level									
Low	1342	1.93 (0.30)	14,806	1.51 (0.10)	9659	1.40 (0.09)	3828	1.95 (0.18)	4.13 *
Moderate	630	1.96 (0.41)	8509	2.69 (0.21)	6930	2.60 (0.15)	2973	2.59 (0.27)	0.42
High	462	5.48 (0.74)	7086	4.75 (0.32)	6472	3.79 (0.18)	2684	3.75 (0.25)	129.46 *
Unclear/unknown	568	2.76 (0.44)	6285	2.19 (0.20)	4116	1.94 (0.19)	1594	2.68 (0.31)	0.07
Education Level									
Low	2424	1.68 (0.28)	29,400	1.48 (0.07)	21,496	1.58 (0.10)	8687	1.83 (0.16)	36.96 *
Moderate	317	3.46 (0.79)	4472	3.95 (0.31)	3722	3.25 (0.21)	1631	3.83 (0.31)	3.80
High	261	5.81 (0.63)	2814	6.25 (0.38)	1959	5.44 (0.29)	761	5.77 (0.38)	8.30 *
Marital Level									
Spinsterhood	297	4.96 (0.65)	1628	6.23 (0.45)	666	4.65 (0.42)	323	4.90 (0.60)	8.23 *
Married/cohabitation	2504	2.12 (0.24)	33,298	2.14 (0.10)	25,363	2.28 (0.11)	10,285	2.46 (0.13)	46.12 *
Widowed/divorce/separation	201	0.19 (0.08)	1760	1.09 (0.15)	1148	1.07 (0.17)	471	2.52 (0.81)	60.30 *
Employment									
Farming and aquaculture	1389	0.59 (0.12)	17,723	0.93 (0.07)	11,690	0.91 (0.06)	4556	1.09 (0.11)	14.54 *
Others	770	4.76 (0.62)	9849	4.89 (0.24)	7317	4.56 (0.16)	2991	5.16 (0.25)	0.28
Student	30	8.03 (2.62)	126	10.47 (1.04)	55	7.10 (1.25)	26	8.14 (2.15)	1.82
Unemployed/retired	813	0.91 (0.25)	8988	0.78 (0.06)	8115	0.97 (0.09)	3506	0.81(0.09)	2.18

Data are presented as $N\;or\;\bar{x}$(s). * *p* < 0.05 compared with Normal, Overweight and Obesity.

**Table A2:** Distribution of frequencies of eating-away-from-home (EAFH) of different sexes and ages in urban and rural areas.

Variable	City				Rural			
	Male		Female		Male		Female	
	*n*	EAFH	*n*	EAFH	*n*	EAFH	*n*	EAFH
Age, years								
18–44	4135	5.20 (0.09)	5243	3.68 (0.07)	6281	2.26 (0.06)	7411	1.15 (0.04)
45–59	4962	3.03 (0.07)	6056	1.60 (0.05)	8315	1.42 (0.04)	9419	0.50 (0.02)
>60	5461	0.94 (0.04)	5708	0.59 (0.03)	7682	0.51 (0.03)	7271	0.19 (0.02)
BMI (kg/m^2^)								
Underweight	377	2.83 (0.26)	580	2.35 (0.18)	926	1.15 (0.11)	1119	0.68 (0.08)
Normal	5820	2.72 (0.06)	7590	2.26 (0.05)	11,645	1.25 (0.03)	11,631	0.67 (0.02)
Overweight	5907	2.89 (0.06)	6042	1.67 (0.05)	7232	1.36 (0.04)	7996	0.56 (0.03)
Obesity	2454	3.14 (0.10)	2795	1.35 (0.06)	2475	1.81 (0.08)	3355	0.46 (0.04)

**Table A3:** Distribution of frequencies of eating-away-from-home (EAFH) of different BMI in urban and rural adults aged 18–44 years.

Variable	City				Rural			
	Male		Female		Male		Female	
	*n*	EAFH	*n*	EAFH	*n*	EAFH	*n*	EAFH
Underweight	141	5.44 (0.52)	291	4.04 (0.30)	288	2.22 (0.27)	423	1.52 (0.19)
Normal	1711	5.03 (0.14)	3024	3.90 (0.10)	3164	2.21 (0.08)	4018	1.22 (0.05)
Overweight	1527	5.14 (0.15)	1364	3.43 (0.14)	1966	2.18 (0.10)	2104	1.07 (0.07)
Obesity	756	5.63 (0.21)	564	2.98 (0.20)	863	2.65 (0.16)	866	0.82 (0.09)
